# *HTT, ATXN1* and *ATXN2* CAG triplet repeat sizes: exploring their role in the disease risk and cancer comorbidity in Parkinson’s disease

**DOI:** 10.1093/braincomms/fcaf060

**Published:** 2025-02-06

**Authors:** Sergio Pérez-Oliveira, Ignacio Álvarez, Manuel Menéndez-González, Israel David Duarte-Herrera, Marta Blázquez-Estrada, Juan Castilla-Silgado, Esther Suárez, Ciara García-Fernández, Pablo Siso-García, Pablo García-González, Maitee Rosende-Roca, Mercè Boada, Agustín Ruiz, Jon Infante, Beatriz De la Casa-Fages, Isabel González-Aramburu, Victoria Álvarez, Pau Pastor

**Affiliations:** Laboratorio de Genética, Hospital Universitario Central de Asturias, 33011 Oviedo, Spain; Instituto de Investigación Sanitaria del Principado de Asturias (ISPA), 33011 Oviedo, Spain; Department of Neurology, Movement Disorders Unit, University Hospital Mútua de Terrassa and Fundació Docència i Recerca Mútua de Terrassa, 08221 Terrassa, Barcelona, Spain; Instituto de Investigación Sanitaria del Principado de Asturias (ISPA), 33011 Oviedo, Spain; Servicio de Neurología, Hospital Universitario Central de Asturias, 33011 Oviedo, Spain; Departamento de Medicina, Universidad de Oviedo, 33006 Oviedo, Spain; Instituto de Investigación Sanitaria del Principado de Asturias (ISPA), 33011 Oviedo, Spain; Networked Biomedical Research Center (CIBER)—Respiratory Diseases, 28029 Madrid, Spain; Instituto de Investigación Sanitaria del Principado de Asturias (ISPA), 33011 Oviedo, Spain; Servicio de Neurología, Hospital Universitario Central de Asturias, 33011 Oviedo, Spain; Departamento de Medicina, Universidad de Oviedo, 33006 Oviedo, Spain; Asociación Parkinson Asturias (APARKAS), 33011 Oviedo, Spain; Instituto de Investigación Sanitaria del Principado de Asturias (ISPA), 33011 Oviedo, Spain; Servicio de Neurología, Hospital Universitario Central de Asturias, 33011 Oviedo, Spain; Instituto de Investigación Sanitaria del Principado de Asturias (ISPA), 33011 Oviedo, Spain; Servicio de Neurología, Hospital Universitario Central de Asturias, 33011 Oviedo, Spain; Instituto de Investigación Sanitaria del Principado de Asturias (ISPA), 33011 Oviedo, Spain; Servicio de Neurología, Hospital Universitario de Cabueñes, 33394 Gijón, Spain; Ace Alzheimer Center Barcelona, Universitat Internacional de Catalunya, 08028 Barcelona, Spain; Ace Alzheimer Center Barcelona, Universitat Internacional de Catalunya, 08028 Barcelona, Spain; Networking Research Center on Neurodegenerative Diseases (CIBERNED), Instituto de Salud Carlos III, 28029 Madrid, Spain; Ace Alzheimer Center Barcelona, Universitat Internacional de Catalunya, 08028 Barcelona, Spain; Networking Research Center on Neurodegenerative Diseases (CIBERNED), Instituto de Salud Carlos III, 28029 Madrid, Spain; Ace Alzheimer Center Barcelona, Universitat Internacional de Catalunya, 08028 Barcelona, Spain; Networking Research Center on Neurodegenerative Diseases (CIBERNED), Instituto de Salud Carlos III, 28029 Madrid, Spain; Networking Research Center on Neurodegenerative Diseases (CIBERNED), Instituto de Salud Carlos III, 28029 Madrid, Spain; Department of Neurology, Marqués de Valdecilla University Hospital, Universidad de Cantabria, 39008 Santander, Spain; Department of Neurology, Movement Disorders Unit, Hospital General Universitario Gregorio Marañón, 28007 Madrid, Spain; Instituto Investigación Sanitaria Gregorio Marañón, 28007 Madrid, Spain; Networking Research Center on Neurodegenerative Diseases (CIBERNED), Instituto de Salud Carlos III, 28029 Madrid, Spain; Department of Neurology, Marqués de Valdecilla University Hospital, Universidad de Cantabria, 39008 Santander, Spain; Laboratorio de Genética, Hospital Universitario Central de Asturias, 33011 Oviedo, Spain; Instituto de Investigación Sanitaria del Principado de Asturias (ISPA), 33011 Oviedo, Spain; Department of Neurology, Unit of Neurodegenerative diseases, University Hospital Germans Trias i Pujol and The Germans Trias i Pujol Research Institute (IGTP) Badalona, 08916 Barcelona, Spain

**Keywords:** Parkinson’s disease, cancer, CAG repeats

## Abstract

Parkinson’s disease genetic embraces genetic and non-genetic factors. It has been suggested a link between CAG repeat number in the *HTT, ATXN1* and *ATXN2* genes and different neurodegenerative diseases. Several genetic factors involved in Parkinson’s disease development are indeed associated with cancer pathways. Moreover, several studies found a low prevalence of cancer in neurodegenerative diseases that can be associated with a low CAG repeat size in several genes. This study aimed to investigate the influence of CAG repeat sizes in *ATXN1, ATXN2* and *HTT* genes on the risk for developing cancer and Parkinson’s disease in a large cohort of patients with idiopathic Parkinson’s disease and healthy controls. The work included 1052 patients with idiopathic Parkinson’s disease and 1070 controls of European ancestry. CAG repeat sizes in *HTT, ATXN1* and *ATXN*2 genes were analysed. Dunn’s multiple comparison test for quantitative variables and logistic and linear regression were used. The long *ATXN1* and *HTT* alleles and CAG size and both the *ATXN2* short and long alleles were predictors for the Parkinson’s disease risk. The long CAG *ATXN1* allele gene was associated with the risk of cancer. No association was observed between CAG size in the *HTT* and *ATXN2* genes and risk of cancer in patients with Parkinson’s disease. We described an association of *HTT, ATXN1* and *ATXN2* with the risk of Parkinson’s disease, which reinforce the hypothesis of the common pathway of neurodegeneration. Besides, *ATXN1* could be a predictor of cancer risk among patients with Parkinson’s disease, and these results suggest that cancer and neurodegeneration processes can share common pathways.

## Teaser

Pérez-Oliveira *et al*. reported association between CAG repeat expansion disorders and the risk of Parkinson disease and also investigated the connection between CAG repeats and cancer comorbidity in patients with Parkinson’s disease. *ATXN1* could be a predictor of cancer risk among patients with Parkinson’s disease, suggesting that cancer and neurodegeneration process can share common pathways.

## Graphical Abstract

**graphical abstract:**
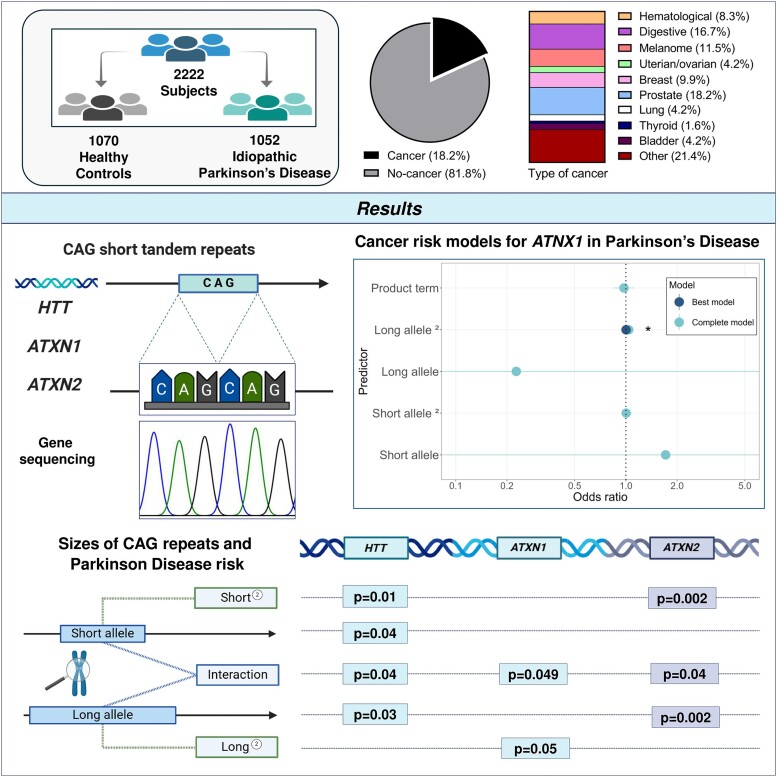

## Introduction

Parkinson’s disease is the second most frequent neurodegenerative disease with an estimated prevalence of about 1% in individuals older than 60 years and 2% of the population older than 65 years. Parkinson’s disease rarely is monogenic, and, most frequently, variants in several genes increase the risk for Parkinson’s disease. Thus, Parkinson’s disease is a complex disease where genetic and non-genetic factors are involved in the aetiology of the disease.^1-3^

Spinocerebellar ataxia type 1 (SCA1), spinocerebellar ataxia type 2 (SCA2) and Huntington disease are autosomal-dominant genetically determined neurodegenerative diseases, caused by the expanded CAG repeats (polyglutamine, polyQ) at the *ATXN1, ATXN2 a*nd *HTT* genes, respectively. PolyQ inclusions accumulate in the cell and interact with several transcription factors causing a dysfunction of the cellular machinery and cell death. The pathological expansions in the *HTT* gene cause upregulation of transcriptional activity of tumour suppressor protein P53. P53, which is highly expressed in patients with Huntington disease, could explain the decreased risk of cancer in this disease. Oppositely, it has been described that a long size of *HTT* CAG repeats is associated with worse cancer outcomes.^4,5^ ATXN2 is an RNA-binding protein that regulates mRNA translation and protein synthesis and participates in the stress response. It is involved in m6A methylation, which is an important event in oncologic pathways. Indeed, ATXN2 overexpression is correlated with the proliferation and metastasis of pancreatic and oesophageal cancer.^6,7^ ATXN1 is a component of the Notch signalling pathway and controls cell proliferation and the epithelial-mesenchymal transition of cancer cells.^8^

On the other hand, the incidence of cancer is decreased in patients with neurodegenerative disorders, including polyQ diseases.^9,10^ Hence, a common mechanism against the development of cancer when certain proteins normally function at the CNS and in other tissues is plausible.

Epidemiological data suggest that patients with Parkinson’s disease have a lower risk of developing cancer compared with the general population; several Parkinson’s disease risk genes have also been shown to play a role in oncogenesis. For example, Lrrk2 is overexpressed in papillary renal and thyroid carcinomas, and Parkin is a tumour suppressor protein involved in a variety of cancers.^11-14^ It has also been reported that increased risk of leukaemia and skin and colon cancer among patients with Parkinson’s disease with *LRRK2* pathogenic variants and *PRKN* has been associated with colorectal tumour progression and lung cancer.^15-19^ In addition, an older Parkinson’s disease onset among idiopathic patients who developed cancer before Parkinson’s disease has been described; alcohol consumption is an important risk factor for developing cancer among patients with Parkinson’s disease, and the TT *GRN*-rs5848 genotype frequency is higher among Parkinson’s disease patients without cancer.^20^

In this work, we explore the relationships between CAG repeats with the susceptibility to develop Parkinson’s disease and the occurrence of cancer comorbidity in a large cohort of idiopathic Parkinson’s disease (iPD) patients.

## Materials and methods

### Subjects

Our cohort consisted of 1052 unrelated consecutive patients who were clinically diagnosed with iPD according to the standard criteria.^21^ A total of 1024 of 1052 patients with Parkinson’s disease were previously genotyped for CAG repeats in the *HTT* gene.^22^ In this work, we included the values of the CAG repeats because a different statistical approach was used, with Cohen’s *d* test as a measure of the strength of the relationship between repeat alleles and Parkinson’s disease. In addition, an analysis of quadratic and product term for the CAG repeat alleles was applied. Moreover, 660 of 1052 patients were used in previous work about cancer risk factor in Parkinson’s disease.^20^ All the participants were of European ancestry and were recruited at the Neurology Units at Hospital Universitario de Cabueñes (Gijón, Spain), Hospital Universitario Central de Asturias (Oviedo, Spain), Hospital General Universitario Gregorio Marañón (Madrid, Spain), Hospital Universitari Mutua Terrassa (Barcelona, Spain) and the Hospital Universitario Marqués de Valdecilla (Santander, Spain). Information regarding sex, age at onset (AAO), age at last assessment, disease duration and death, history of cancer, type, AAO of cancer and alcohol and tobacco consumption was collected from the patients’ clinical records. No data about family history of cancer were available. Thus, the cancer risk study was carried out only taking into account the Parkinson’s disease cohort.

Besides, we have a control group that consists of individuals with European ancestry, free of neurodegenerative diseases who agreed to join the study (*n* = 1070). They were recruited through the Health Community Service of Asturias (Oviedo, Spain) and Alzheimer Center (Barcelona, Spain). This control group was used to estimate the association between the risk of developing Parkinson’s disease and the CAG triplet sizes.

All the participants gave their informed consent to participate in the study, approved by the Ethical Committees (approval ID: CEImPA 2020.333).

### Genetic analysis

The genomic DNA was isolated from peripheral blood leucocytes following standard procedures. As described in previous studies,^20^ the DNA samples were analysed using a next-generation sequencing gene panel that included the most frequent genes linked to monogenic Parkinson’s disease (Supplementary Table 1). Patients without any pathogenic variant were considered as having iPD and were included in the study.

*HTT, ATXN1* and *ATXN2* gene CAG repeats sizes were analysed by a polymerase chain reaction with 5′-fluorescence labelled primers. To determine the size of the HTT repeats, we used the triplet repeat primed PCR. The number of CAG repeats was determined by capillary electrophoresis using an ABI 3130X automated DNA sequencer and the GeneMapper version 4.0 software (Applied Biosystems, Foster City, CA, USA). To obtain size standards, several samples with validated different *HTT, ATXN1* and *ATXN2* CAG allele lengths were sequenced. These samples included carriers of normal, intermediate and expanded alleles (Supplementary Fig. 1). We genotyped the CAG repeats in the *ATXN1* and *ATXN2* genes in all Parkinson’s disease and healthy controls, while for *HTT* CAG repeats, the majority of the patients (1024) and all the controls had been genotyped in a previous work.^22^ Ranges for allele HTT CAG expansion categories were established as follows: normal alleles <27, intermediate alleles (IAs) ≥27 and ≤35, expanded alleles ≥36; for ATXN1 CAG repeats: normal alleles <33; IAs ≥33 and ≤38 and expanded alleles >38; for ATXN2 CAG repeats: normal alleles <27, IAs ≥ 27 and ≤33 and expanded alleles ≥33.

### Statistical analyses

Categorical variables (normal, intermediate or expanded *HTT*, *ATXN1* and *ATXN2* CAG alleles, history of cancer, sex, drinking and smoking) were described in frequencies and percentages. To study the frequencies of these variables, Chi-squared and Fisher’s exact test were carried out. Holm–Bonferroni method was used for multiple comparison *post hoc* corrections. Mean and standard deviation were used for the analysis of continuous variables (*HTT, ATXN1* and *ATXN2* CAG repeat size, AAO, age at last assessment/death and disease duration). Dunn’s multiple comparison test with Holm correction was performed for quantitative variables after using Kruskal–Wallis Test. Wilcoxon test was also performed for quantitative variables with two groups. For the Wilcoxon test, Cohen’s *d* is calculated for measure effect size.^23^

Multiple linear regression models and binary logistic regression models were used to explore the interaction between genetic and non-genetic variables, the risk of Parkinson’s disease and the risk for cancer and the age at disease onset in patients with Parkinson’s disease. Stepwise regression using the Akaike Information Criterion (AIC) was applied to optimize the models. For all comparisons performed, a *P*-value of <0.05 suggests that there is sufficient evidence to reject the null hypothesis. Analyses were performed using R software version 4.3.2.

## Results

### Demographic data

A total of 1052 iPD patients and 1070 healthy controls were analysed. Data on cancer prevalence and type of cancer were available in 94% of the patients (*n* = 990), among which, 18.2% (*n* = 180) suffered cancer (Table 1; Supplementary Fig. 2). Among cancer patients, prostate cancer was the most common.

**Table 1:** Summary of demographic and clinical characteristics for all patients with Parkinson’s disease

Variables	iPD			Controls
	All Parkinson’s disease	Parkinson’s disease with cancer^c^	Parkinson’s disease without cancer^c^	
*N* (%)	1052	180 (18.2%)	810 (81.8%)	1070
AAO	62.15 ± 11.63	65.82 ± 9.88	61.16 ± 11.73	
Duration	9.84 ± 7.36	8.79 ± 6.33	9.82 ± 6.65	
Age at last assessment	71.69 ± 11.25	74.6 ± 8.65	70.7 ± 11.42	71.09 ± 7.97
Sex (male)	614 (58.4%)	121 (67.2%)	464 (57.3%)	494 (46.2%)
Smoking^a^	73 (13.3%)	15 (19.5%)	56 (12.1%)	16 (5.2%)
Drinking^b^	42 (7.7%)	12 (15.6%)	28 (6.0%)	

All values are expressed as mean ± SD or *n* (%).

^a^Smoking data available in 549 patients (52.2%) and 306 controls (28.8%).

^b^Drinking data available in 549 patients (52.2%).

^c^Cancer data available in 990 patients (94.1%). Two patients with smoking and drinking data do not have cancer data.

In respect to the distribution of sex, a clear increase in the frequency of male in Parkinson’s disease compared with controls {58.5 versus 46.2%; *P* = 2.01e−08, odds ratio [OR] [95% confidence interval (CI)]: 1.63 [1.37–1.95]} was observed. Concerning the risk of cancer and according to our previous results, in the sub-cohort of 660 iPD, a higher age at Parkinson’s disease onset (68.87 ± 9.65) was observed in patients who developed cancer before Parkinson’s disease compared with patients without cancer (*P* = 2.1e−04). Alcohol consumption was a risk factor to develop cancer (15.6 versus 6.0% no cancer Parkinson’s disease) in patients with Parkinson’s disease [*P* = 7.58e−03, OR (95% CI): 2.86 (1.26–6.16)], while no differences were observed between smokers and non-smokers^20^ (Table 1).

### Frequencies of normal, intermediate and expanded HTT, ATXN1 and ATXN2 alleles

**Figure 1:**
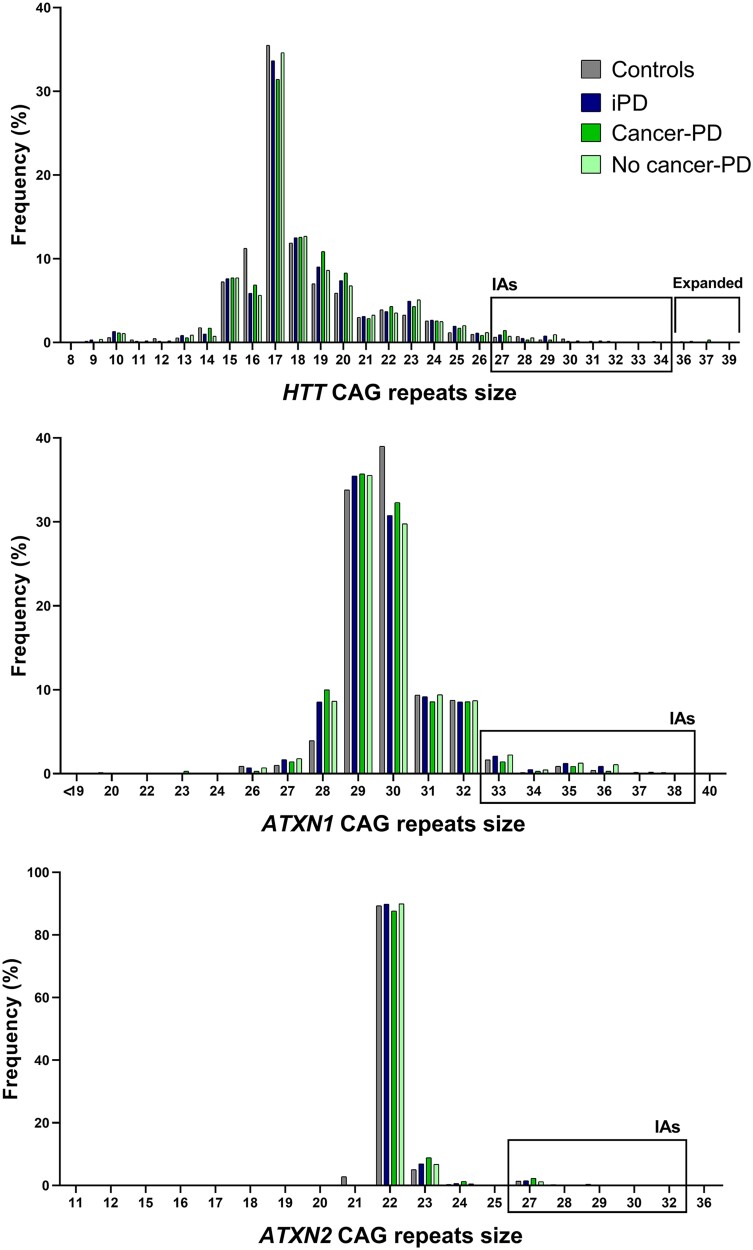
**Distribution of *HTT*, *ATXN1* and *ATXN2* CAG alleles across the different groups.** The graphs show the relative frequency (%) of different CAG repeat sizes in the *HTT*, *ATXN1* and *ATXN2* genes across four groups: controls, iPD, Parkinson’s disease associated with cancer (cancer-PD) and Parkinson’s disease not associated with cancer (no cancer-PD). For the *HTT* gene, IAs range from 27 to 35 repeats, and expanded alleles exceed 36 repeats. In the *ATXN1* gene, IAs are defined as 35–39 repeats. For the *ATXN2* gene, IAs correspond to 27–32 repeats. Each data point represents the percentage of individuals within each group carrying a given repeat size.

The analysis of the whole sample showed that the most frequent allele sizes were 17 and 18 CAG repeats at the *HTT* gene, 22 CAG repeats at *ATXN2* and 29–30 CAG repeats at *ATXN1* (Fig. 1). The frequency of different alleles was similar to those reported in other European populations.^24^ The statistical test showed that patients with Parkinson’s disease and controls had a similar frequency distribution for normal range and IAs in *HTT* and *ATXN2* genes among patients with Parkinson’s disease and controls (Table 2). For the *ATXN1* gene, we observed an increased frequency of IAs in patients with Parkinson’s disease compared with controls, but it was not significant [9.5 versus 6.3%, respectively; *P* = 0.576, OR (95% CI): 0.64 (0.39–1.02)].

**Table 2:** Frequencies of IAs in the *HTT*, *ATXN1* and *ATXN2* genes in Parkinson’s disease cohorts and healthy controls

Group	HTT IAs carriers	*HTT* expanded carriers^a^	Holm-corrected *P*-value^d^ [OR (95% CI)]	*ATXN1* IAs carriers	*ATXN1* expanded carriers	Holm-corrected *P*-value^d^ [OR (95% CI)]	*ATXN2* IAs carriers	*ATXN2* expanded carriers	Holm-corrected *P*-value^d^ [OR (95% CI)]
Parkinson’s disease^b^	52 (5.0)	3 (0.3)	1.0 [0.81 (0.52–1.25)]	97 (9.5)	0	0.576 [0.64 (0.39–1.02)]	33 (3.7)	0	0.593 [1.18 (0.68–2.05)]
Controls	43 (4.1)	2 (0.2)		25 (6.3)	0		27 (4.3)	0	
iPD cohort									
Parkinson’s disease with cancer^c^	7 (3.9)	1 (0.6)	1.0 [1.39 (0.61–3.72)]	10 (5.7)		0.064 [1.91 (0.96–4.23)]	6 (3.8)		0.812 [0.91 (0.36–2.77)]
Parkinson’s disease without cancer	43 (5.3)	2 (0.2)		81 (10.4)			24 (3.5)		

All values are expressed as *n* (%). Fisher *t*-test with Holm correction.

^a^Results found in a previous work.^22^

^b^Comparisons versus controls.

^c^Comparisons of patients with cancer versus patient without cancer.

^d^The *P*-value correspond to the comparison of the frequency of IAs in patients versus healthy controls and the comparison of Parkinson’s disease patients with cancer versus Parkinson’s disease patients without cancer.

Neither A*TXN1* nor *ATXN2* expanded alleles were detected. Three patients carrying interrupted low penetrance *HTT* expanded alleles were detected in our previous work.^22^

When we stratified the Parkinson’s disease cohort by cancer and non-cancer, no differences in the allele distribution in the *HTT* and *ATXN2 g*ene were observed. However, we observed a non-significant increased frequency of *ATXN1* IAs in the non-cancer patients with Parkinson’s disease compared with the cancer controls [10.4 versus 5.7%; *P* = 0.19, OR (95% CI): 1.91 (0.961–4.23)].

### HTT, ATXN1 and ATXN2 CAG repeat sizes

#### CAG repeat sizes in the different cohorts

A case–control or cancer–non-cancer Parkinson’s disease comparisons were conducted to examine the size of the *HTT, ATXN1* and *ATXN2* CAG repeats in the different groups. A difference in both alleles of the *HTT* gene compared with the controls was observed: the short allele (16.83 ± 2.24 versus 16.68 ± 2.05; *P* = 8.86e−04; *d* = 0.07) and the long allele (20.15 ± 3.40 versus 19.67 ± 3.40; *P* = 6.1e−05; *d* = 0.14). A similar result was observed at the *ATXN2* gene in both, the short allele (21.96 ± 0.59 versus 21.94 ± 0.34; *P* = 1.36e−05; *d* = 0.05) and the long allele (22.38 ± 1.27 versus 22.35 ± 1.21; *P* = 0.038; *d* = 0.017). For the *ATXN1* gene, differences were only observed in the short allele (29.04 ± 1.08 versus 29.21 ± 1.01; *P* = 3.9e−04; *d* = 0.16). Although there were significant differences, the effect size was practically negligible according to Cohen’s ‘*d*’ (*d*) (Table 3).

**Table 3:** *HTT*, *ATXN1* and *ATXN2* CAG repeat sizes and risk of disease and cancer occurrence in patients with Parkinson’s disease

	Short allele						Long allele					
Group	*HTT* repeat size	*P*-value (effect size)	*ATXN1* repeat size	*P*-value (effect size)	*ATXN2* repeat size	*P*-value (effect size)	*HTT* repeat size	*P*-value (effect size)	*ATXN1* repeat size	*P*-value (effect size)	*ATXN2* repeat size	*P*-value (effect size)
iPD	16.83 ± 2.24	**8.86e−04** (0.07)^a^	29.04 ± 1.08	**3.95e−04** (0.16)^a^	21.96 ± 0.59	**1.38e−05** (0.05)^a^	20.15 ± 3.40	**6.07e−05** (0.14)^a^	30.66 ± 1.59	0.73 (0.036) ^a^	22.36 ± 1.19	**0.04** (0.005) ^a^
Controls	16.68 ± 2.05		29.21 ± 1.01		21.94 ± 0.34		19.67 ± 3.40		30.60 ± 1.34		22.35 ± 1.21	
Parkinson’s disease cohort												
Parkinson’s disease with cancer	16.87 ± 2.00	0.96 (0.02)^a^	29.02 ± 1.01	0.64 (0.02)^a^	22.04 ± 0.41	**0.016** (0.17)^a^	20.05 ± 3.26	0.94 (0.04)^a^	30.46 ± 1.35	0.14 (0.16)^a^	22.41 ± 1.03	0.16 (0.0.05)^a^
Parkinson’s disease without cancer	16.84 ± 2.23		29.04 ± 1.04		21.94 ± 0.64		20.18 ± 3.45		30.71 ± 1.65		22.35 ± 1.22	

Bold values: Wilcoxon test with *P*-value <0.05. The magnitude is assessed using the thresholds provided in the study by Cohen,^23^ i.e. |*d*| < 0.2 ‘negligible’, |*d*| < 0.5 ‘small’, |*d*| < 0.8 ‘medium’, otherwise ‘large’.

^a^Cohen’s *d* is added to measure effect size.

After stratifying the patients with Parkinson’s disease according to whether they had cancer or not, we only found that Parkinson’s disease patients with cancer had a statistically significant increase in the length of *ATXN2* CAG repeats compared with Parkinson’s disease without cancer for the short allele (22.04 ± 0.41 versus 21.94 ± 0.64; *P* = 0.016; *d* = 0.17). Although there were significant differences in *ATXN2* length, a small effect size was observed according to Cohen’s ‘*d*’ (*d*).

#### CAG repeats and the risk of disease and cancer occurrence

To study the risk of Parkinson’s disease and the risk of cancer in patients with Parkinson’s disease, binomial logistic models were created considering all variables: sex, drinking, smoking and *HTT, ATXN1* and *ATXN2* CAG repeat sizes. We generated models using the CAG repeat sizes *HTT*, *ATXN*1 and *ATXN2*, the quadratic term for each allele, the product term of the two alleles and finally the presence of the IAs as explanatory variables.^25,26^ The quadratic term for each allele allows us to explore the non-linear relationships between the CAG repeat size of the alleles and Parkinson’s disease risk or cancer risk. The product term of the two alleles was run to investigate possible interaction effects between the two alleles.

**Figure 2:**
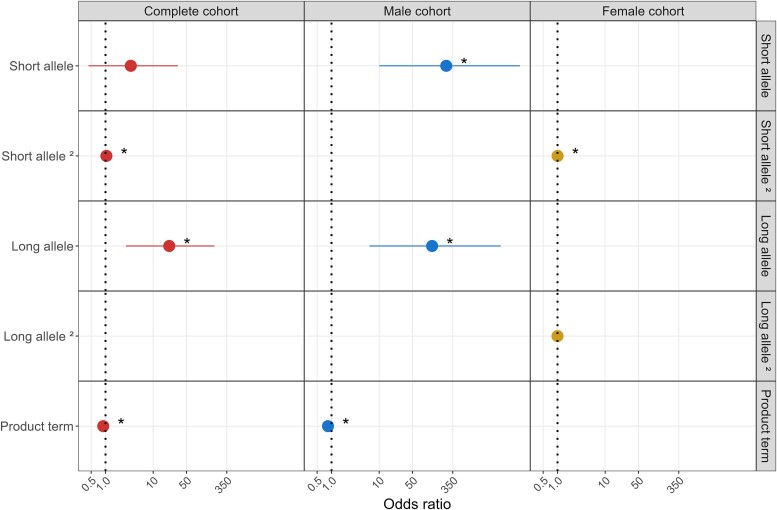
***ATXN1* CAG repeats and Parkinson’s disease risk.** Binomial regression models for Parkinson’s disease risk in the complete cohort and stratified by sex, represented as ORs. *n* = 1504 for the complete cohort, *n* = 817 males, *n* = 687 females. Each point in the graph represents the calculated OR, and the horizontal lines indicate the 95% CIs. ‘Short allele’ and ‘long allele’ refer to the categories of CAG repeat lengths in the *ATXN1* gene; ‘product term’ represents the interaction term; ‘2’ indicates the quadratic term. Statistical test: binomial regression model. *P* < 0.05 (indicated with an asterisk *).

The binomial logistic model that was generated in order to analyse the effect of CAG repeats in the *HTT*, *ATXN1* and *ATXN2* genes on the risk to develop Parkinson’s disease displayed a model with the best prediction performance (*P* = 3.54e−10, AIC = 1181.9) that included the following predictors: linear and quadratic term of the short *HTT* allele (*P* = 0.044 and *P* = 9.95e−03, respectively), long *HTT* allele (*P* = 0.030), quadratic term of the long *ATXN1* allele (*P* = 0.0499), quadratic term of the short *ATXN2* allele (*P* = 0.002); *ATXN2* long allele (*P* = 0.002) and the interaction of both alleles of *HTT* (*P* = 0.039) and *ATXN2* (*P* = 7.3e−05). The best association between the risk of Parkinson’s disease and *HTT* CAG repeats was driven by a model (*P* = 2.43e−04, AIC = 2707.3) in which the risk predictor variable was the long *HTT* allele, both for the linear (*P* = 0.004) and the quadratic terms (*P* = 0.01). After stratifying the cohorts (patients and controls) by sex, the effect of the long *HTT* allele was only sustained among the male model (*P* = 0.03; AIC = 1368.8) when predicting Parkinson’s disease risk. For *ATXN1* gene CAG repeats size, the best logistic model (*P* = 0.001, AIC = 1666.1) included the quadratic term of short allele (*P* = 0.024), the long allele (0.021) and the interaction between both alleles (*P* = 0.017) as predictors for the Parkinson’s disease risk. After stratifying the cohort by sex, the best male model (AIC = 791.4) was included as predictor, both the short (*P* = 0.005) and the long (*P* = 0.008) *ATXN1* allele, as well as its interaction (*P* = 0.007). In the female model (AIC = 809.7), only the quadratic term of the short *ATXN1* allele (*P* = 0.009) showed significance (Fig. 2). The best logistic model for prediction of Parkinson’s disease risk in reference to the *ATXN2* CAG repeats (*P* = 1.68–06; AIC = 2037) included as predictors the quadratic term of the short allele (*P* = 2.72e−04), the long allele (*P* = 0.002) and the interaction between both alleles (*P* = 0.002). No model showed *ATXN2* predictors when dividing the cohort based on sex (Supplementary Table 2).

**Figure 3:**
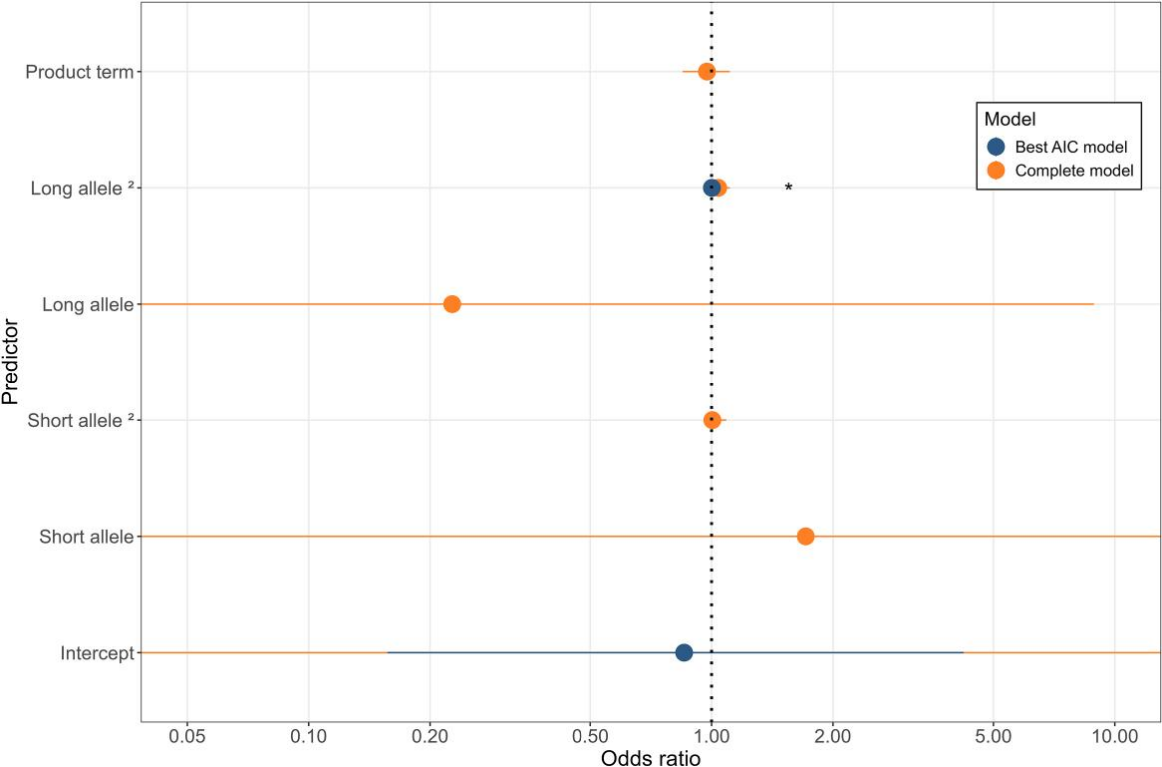
***ATXN1* CAG repeats and cancer risk in Parkinson’s disease patients.** Binomial regression model predictors for cancer risk represented as ORs. *n* = 956. Each point in the graph represents the calculated OR and the horizontal lines indicate the 95% CIs. ‘Short allele’ and ‘long allele’ refer to the categories of CAG repeat lengths in the *ATXN1* gene; ‘2’ indicates the quadratic term; ‘product term’ represents the interaction term for both alleles; ‘intercept’ corresponds to the intercept term of the model. Statistical test: binomial regression model. *P* < 0.05 (indicated with an asterisk *).

Addressing the cancer risk in patients with Parkinson’s disease, being a drinker (*P* = 0.004; AIC = 349.8) seemed to be a risk factor for the cancer comorbidity in patients with Parkinson’s disease, which was a result also reported previously by our group.^20^ Considering the three candidate genes, the best logistic model (*P* = 0.041; AIC = 909.93) was found for the *ATXN1* gene, which showed a statistically significant prediction value of the quadratic term for the long allele [*P* = 0.0491, OR (95% CI): 1.002 (1.00–1.004)] in modifying cancer risk among patients with Parkinson’s disease (Fig. 3). Neither the *HTT* gene nor the *ATXN2* gene showed a significant predictive effect on cancer risk in patients with Parkinson’s disease.

Finally, to evaluate whether *HTT, ATXN1* and *ATXN2* CAG repeat sizes were associated with age at disease onset, a linear regression model was generated using age of onset as the independent variable. Independent quadratic models for each gene, including the presence/absence of IAs and their length, also did not provide relevant associations with disease onset.

## Discussion

In this study, we investigated the effect of *HTT, ATXN1* and *ATXN2* CAG allele sizes in the risk of developing the disease and cancer comorbidity in a large cohort of patients with iPD.

### CAG repeats and Parkinson’s disease risk

The long *ATXN1 a*llele and the CAG size and both the short and long alleles of *ATXN2* were predictor variables of the Parkinson’s disease risk. In addition, we confirmed the long *HTT* allele, both linear and quadratic terms, as a disease risk predictor among males with Parkinson’s disease.

Statistical differences in the mean of the CAG repeats were observed between patients with Parkinson’s disease and controls and between cancer and non-cancer Parkinson’s disease patients. However, it is important to emphasize that the small effect size, as reflected by Cohen’s ‘*d*’, indicates that the practical relevance of this finding is limited. This is a common occurrence in studies with large sample sizes, where even small differences can reach statistical significance due to reduced sample variance.

Neurodegenerative diseases accumulate different protein aggregates in the brain, such as amyloid, tau, α-synuclein (a-syn) or TAR DNA-binding protein 43,^27-31^ though very often different protein aggregates are present within the same individual. A-syn brain aggregates are the main inclusions found in Parkinson’s disease brains, whereas huntingtin (Htt), ataxin-1 and ataxin-2 are the main components of Huntington disease, SCA1 and SCA2, respectively. We previously reported a link between *HTT* IAs and an α-synucleinopathy called multisystem atrophy and the presence of expanded *HTT* CAG repeats in three patients with Parkinson’s disease, thus supporting a possible role of Htt on Parkinson’s disease aetiology.^22^ However, the association of the *ATXN1* and *ATXN2* with risk for Parkinson’s disease could be difficult to explain, although an association of *ATXN2* and the *ATXN1* CAG repeats with Alzheimer’s disease, frontotemporal dementia and amyotrophic lateral sclerosis has been described.^24,32^

Ataxin-1 is a protein with polyglutamine stretches that causes SCA1 through a toxic gain-of-function that involves nuclear aggregation of *ATXN1* protein, and it has been described as the inter-cellular propagation of polyglutamine-expanded *ATXN1* inclusions.^33^ Inter-cellular propagation of aggregated protein inclusions along actin-based tunnelling nanotubes has been reported as a mean of pathogenic spread in Alzheimer’s, Parkinson’s and Huntington’s diseases.^34^ Thus, we hypothesize that ataxin-1 could trigger the seeding propagation of other molecules such as a-syn. With regards to the risk of *ATXN2* for Parkinson’s disease, it has been reported that *ATXN2* repeat expansions have been found in families with autosomal-dominant parkinsonism and in patients with variable phenotypes, including levodopa-responsive early-onset Parkinson’s disease and a history of progressive cerebellar ataxia (31/41 CAG).^35^ Moreover, previous work has been showed that in cellular models the *ATXN2* allele replacement with a pathogenic 58-repeats *ATXN2* CAG expansion does induce the expression of a-syn.^36^ These data support the association of the polyQ repeats length with Parkinson’s disease but further studies in other patient cohorts are needed to confirm this hypothesis.

Future biochemical studies are needed to elucidate whether HTT*, ATXN1* and *ATXN2* CAG can trigger not only the Htt or ataxin aggregation but also other pathological proteins such as a-syn as well, and this explains their Parkinson’s disease genetic risk.

### CAG repeats and risk of cancer in patients with Parkinson’s disease

We found that CAG repeat number variations in the long *ATXN1* gene were associated with the risk of cancer, an important relationship from both biological and clinical point of view. When we stratified by cancer and non-cancer, we observed a non-statistically significant increased frequency of *ATXN1* IAs in the Parkinson’s disease non-cancer group compared with cancer group controls (10.4 versus 5.7%). No association between Parkinson’s disease risk cancer and the size of CAG repeats on the *HTT* and *ATXN2* genes was observed. Ataxin-1 loss-of-function has been implicated in cancer development probably owing to its role in the cell proliferation and in the epithelial-mesenchymal transition of cervical cancer cells.^8^ In addition, ataxin-1 can enhance E-cadherin expression in the Michigan Cancer Foundation-7 breast cancer cell line and E-cadherin mRNA levels are higher in presence of mutant ataxin-1 (82Q) than with wild-type ataxin-1 (30Q)^37^ Subsequently, the inhibition of E-cadherin expression is regarded as one of the main molecular events responsible for dysfunction in cell–cell adhesion, which can lead to local invasion and, ultimately, to tumour development.^38,39^ Thus, it is possible that long *ATXN1* CAG repeats, even in the normal range, could affect the expression levels of different molecules involved in cancer networks.

Our impression is that the frequency of cancer in our Parkinson’s disease cohort (18.2%) is similar to that described in other studies focused on Spanish Parkinson’s disease cohorts.^40^ The most prevalent types of cancer in our cohort were prostate cancers followed by digestive cancer and skin cancer. These results agree with other studies that identified a positive correlation of Parkinson’s disease with cancer and melanoma.^41^ However, in this work due to the small numbers of patients with cancer, it was not possible to analyse the CAG repeat size among the different cancer kinds.

In recent years, several pieces of evidence suggest that there are many common molecular pathways between cancer and neurodegenerative diseases, including deregulation of apoptosis, autophagy and oxidative stress conditions.^42-44^ Some studies revealed that the cancer prevalence in polyQ diseases is lower than in the general population. Thus, a decrease in the incidence of most cancer types in individuals with Huntington disease or other polyglutamine disorders, including SCA, has been described.^9,45^

We are aware that our study has several limitations. The most important are the variability of the available data (alcohol and smoking occurrence of cancer) because they were collected retrospectively, and no data were available for some of the participants. Also, the sample size of patients with Parkinson’s disease with cancer was small. After incorporating both linear and quadratic terms, as well as allele interaction effects in the statistical analysis of CAG repeats alleles, we captured complex non-linear relationships that revealed new insights beyond traditional linear approaches. However, the cancer risk model demonstrated only a marginal association with the *ATXN1* gene, suggesting a minimal effect size that warrants cautious interpretation and limited use as an independent predictor. A replication of our results in additional series of patients and controls is necessary to confirm the robustness of our results and their potential clinical application.

## Conclusion

To our knowledge, this is the first time that an association between *ATXN1* and *ATXN2* genes with the risk to develop Parkinson’s disease is described. The association with *HTT* CAG repeats has been previously described by our group^22^ and, in the present study, is confirmed in a cohort slightly larger in size using a more accurate statistical analysis, which included a product term of both alleles and a quadratic term for each allele as fixed effects. Our work is also focused on studying the relationship between CAG repeats and the risk of cancer in patients with Parkinson’s disease, another novel research line not previously explored.

Finally, these results reinforce the hypothesis that neurodegenerative diseases could share common pathways, which could have a connection with cancer networks. Thus, it might be possible to speculate about the discovery of new therapeutic strategies for neurodegeneration based on some cancer treatments. In fact, recently it has been described that the inhibition of indoleamine-2,3-dyoxigenase 1, used in cancer therapy, improves cognition in pre-clinical models of amyloid and tau pathology.^46^

## Supplementary Material

fcaf060_Supplementary_Data

## Data Availability

The data and statistics that support the findings of this study are available in Github repository: https://github.com/sergio30po/PD_cancer.git.

## References

[1] Ascherio A , Schwarzschild MA. The epidemiology of Parkinson’s disease: Risk factors and prevention. Lancet Neurol. 2016;15(12):1257–1272. 27751556 10.1016/S1474-4422(16)30230-7

[2] Koros C , Simitsi A, Stefanis L. Genetics of Parkinson’s disease: Genotype–phenotype correlations. Int Rev Neurobiol. 2017;132:197–231. 28554408 10.1016/bs.irn.2017.01.009

[3] Paul KC , Chuang YH, Shih IF, et al The association between lifestyle factors and Parkinson’s disease progression and mortality. Mov Disord. 2019;34(1):58–66. 30653734 10.1002/mds.27577 PMC6544143

[4] Song YN , Geng JS, Liu T, et al Long CAG repeat sequence and protein expression of androgen receptor considered as prognostic indicators in male breast carcinoma. PLoS One. 2012;7(12):e52271. 23272232 10.1371/journal.pone.0052271 PMC3522691

[5] Thion MS , Tézenas Du Montcel S, Golmard JL, et al CAG repeat size in huntingtin alleles is associated with cancer prognosis. Eur J Hum Genet. 2016;24(9):1310–1315. 26980106 10.1038/ejhg.2016.13 PMC4989202

[6] Fang L , Wang SH, Cui YG, Huang L. LINC00941 promotes proliferation and metastasis of pancreatic adenocarcinoma by competitively binding miR-873-3p and thus upregulates ATXN2. Eur Rev Med Pharmacol Sci. 2021;25(4):1861–1868. 33660796 10.26355/eurrev_202102_25081

[7] Li R , Zeng L, Zhao H, et al ATXN2-mediated translation of TNFR1 promotes esophageal squamous cell carcinoma via m6A-dependent manner. Mol Ther. 2022;30(3):1089–1103. 34995801 10.1016/j.ymthe.2022.01.006 PMC8899599

[8] Kang AR , An HT, Ko J, Kang S. Ataxin-1 regulates epithelial-mesenchymal transition of cervical cancer cells. Oncotarget. 2017;8(11):18248–18259. 28212558 10.18632/oncotarget.15319 PMC5392324

[9] Coarelli G , Diallo A, Thion MS, et al Low cancer prevalence in polyglutamine expansion diseases. Neurology. 2017;88(12):1114–1119. 28202696 10.1212/WNL.0000000000003725

[10] Plun-Favreau H , Lewis PA, Hardy J, Martins LM, Wood NW. Cancer and neurodegeneration: Between the devil and the deep blue sea. PLoS Genet. 2010;6(12):e1001257. 21203498 10.1371/journal.pgen.1001257 PMC3009676

[11] Looyenga BD , Furge KA, Dykema KJ, et al Chromosomal amplification of leucine-rich repeat kinase-2 (LRRK2) is required for oncogenic MET signaling in papillary renal and thyroid carcinomas. Proc Natl Acad Sci U S A. 2011;108(4):1439–1444. 21220347 10.1073/pnas.1012500108 PMC3029686

[12] Liu J , Zhang C, Hu W, Feng Z. Parkinson’s disease-associated protein Parkin: An unusual player in cancer. Cancer Commun. 2018;38(1):40. 10.1186/s40880-018-0314-z PMC6020249 29941042

[13] Perwez A , Wahabi K, Rizvi MA. Parkin: A targetable linchpin in human malignancies. Biochim Biophys Acta Rev Cancer. 2021;1876(1):188533. 33785381 10.1016/j.bbcan.2021.188533

[14] Xu L , Lin DC, Yin D, Koeffler HP. An emerging role of PARK2 in cancer. J Mol Med. 2014;92(1):31–42. 24297497 10.1007/s00109-013-1107-0

[15] Agalliu I , Ortega RA, Luciano MS, et al Cancer outcomes among Parkinson’s disease patients with leucine rich repeat kinase 2 mutations, idiopathic Parkinson’s disease patients, and nonaffected controls. Mov Disord. 2019;34(9):1392–1398. 31348549 10.1002/mds.27807 PMC6754269

[16] Perwez A , Wahabi K, Kamarudheen S, et al Association of Parkin with P53 expression and their prognostic significance in North Indian colorectal cancer patients. Gene. 2022;33:201029.

[17] Tiwari RR , Wahabi K, Perwez A, et al Implication of alterations in Parkin gene among North Indian patients with colorectal cancer. Turk J Gastroenterol. 2020;31(3):211–220. 32343233 10.5152/tjg.2020.18823 PMC7197922

[18] Picchio MC , Martin ES, Cesari R, et al Alterations of the tumor suppressor gene Parkin in non-small cell lung cancer. Clin Cancer Res. 2004;10(8):2720–2724. 15102676 10.1158/1078-0432.ccr-03-0086

[19] Cesari R , Martin ES, Calin GA, et al Parkin, a gene implicated in autosomal recessive juvenile parkinsonism, is a candidate tumor suppressor gene on chromosome 6q25-q27. Proc Natl Acad Sci U S A. 2003;100(10):5956–5961. 12719539 10.1073/pnas.0931262100 PMC156308

[20] Rosas I , Morís G, Coto E, et al Cancer in Parkinson’s disease: An approximation to the main risk factors. Neurodegener Dis. 2021;21(1–2):36–41. 34673649 10.1159/000520301

[21] Hughes AJ , Daniel SE, Kilford L, Lees AJ. Accuracy of clinical diagnosis of idiopathic Parkinson’s disease: A clinico-pathological study of 100 cases. J Neurol Neurosurg Psychiatry. 1992;55(3):181–184. 1564476 10.1136/jnnp.55.3.181 PMC1014720

[22] Pérez-Oliveira S , Álvarez I, Rosas I, et al Intermediate and expanded HTT alleles and the risk for α-synucleinopathies. Mov Disord. 2022;37(9):1841–1849. 35852957 10.1002/mds.29153

[23] Cohen J . Statistical power analysis. Curr Dir Psychol Sci. 1992;1(3):98–101.

[24] Gardiner SL , Boogaard MW, Trompet S, et al Prevalence of carriers of intermediate and pathological polyglutamine disease-associated alleles among large population-based cohorts. JAMA Neurol. 2019;76(6):650–656. 30933216 10.1001/jamaneurol.2019.0423 PMC6563569

[25] Gardiner SL , Van Belzen MJ, Boogaard MW, et al Large normal-range TBP and ATXN7 CAG repeat lengths are associated with increased lifetime risk of depression. Transl Psychiatry. 2017;7(6):e1143. 28585930 10.1038/tp.2017.116 PMC5534943

[26] Gardiner SL , Van Belzen MJ, Boogaard MW, et al Huntingtin gene repeat size variations affect risk of lifetime depression. Transl Psychiatry. 2017;7(12):1277. 29225330 10.1038/s41398-017-0042-1 PMC5802693

[27] Axenhus M , Winblad B, Tjernberg LO, Schedin-Weiss S. Huntingtin levels are elevated in hippocampal post-mortem samples of Alzheimer’s disease brain. Curr Alzheimer Res. 2020;17(9):858–867. 33272184 10.2174/1567205017666201203125622

[28] Blum D , Herrera F, Francelle L, et al Mutant huntingtin alters tau phosphorylation and subcellular distribution. Hum Mol Genet. 2015;24(1):76–85. 25143394 10.1093/hmg/ddu421

[29] Fernández-Nogales M , Cabrera JR, Santos-Galindo M, et al Huntington’s disease is a four-repeat tauopathy with tau nuclear rods. Nat Med. 2014;20(8):881–885. 25038828 10.1038/nm.3617

[30] Sanford AM . Lewy body dementia. Clin Geriatr Med. 2018;34(4):603–615. 30336990 10.1016/j.cger.2018.06.007

[31] Gao J , Wang L, Huntley ML, Perry G, Wang X. Pathomechanisms of TDP-43 in neurodegeneration. J Neurochem. 2018;176:7–20. 10.1111/jnc.14327 PMC6110993 29486049

[32] Rosas I , Martínez C, Clarimón J, et al Role for ATXN1, ATXN2, and HTT intermediate repeats in frontotemporal dementia and Alzheimer’s disease. Neurobiol Aging. 2020;87:139.e1–139.e7. 10.1016/j.neurobiolaging.2019.10.017 31810584

[33] Huang H , Toker N, Burr E, et al Intercellular propagation and aggregate seeding of mutant ataxin-1. J Mol Neurosci. 2022;72(4):708–718. 34826062 10.1007/s12031-021-01944-1 PMC8986690

[34] Lagalwar S . Mechanisms of tunneling nanotube-based propagation of neurodegenerative disease proteins. Front Mol Neurosci. 2022;15:957067. 35909452 10.3389/fnmol.2022.957067 PMC9336677

[35] Ibanez K , Polke J, Hagelstrom RT, et al Whole genome sequencing for diagnosis of neurological repeat expansion disorders. Lancet Neurol. 2022;21:234–245. 35182509 10.1016/S1474-4422(21)00462-2 PMC8850201

[36] Gandelman M , Dansithong W, Kales SC, et al The AKT modulator A-443654 reduces α-synuclein expression and normalizes ER stress and autophagy. J Biol Chem. 2021;297(4):101191–101191. 34520759 10.1016/j.jbc.2021.101191 PMC8482485

[37] Lee S , Hong S, Kim S, Kang S. Ataxin-1 occupies the promoter region of E-cadherin in vivo and activates CtBP2-repressed promoter. Biochim Biophys Acta Mol Cell Res. 2011;1813(5):713–722. 10.1016/j.bbamcr.2011.01.035 21315774

[38] Mendonsa AM , Na TY, Gumbiner BM. E-cadherin in contact inhibition and cancer. Oncogene. 2018;37(35):4769–4780. 29780167 10.1038/s41388-018-0304-2 PMC6119098

[39] Jeanes A , Gottardi CJ, Yap AS. Cadherins and cancer: How does cadherin dysfunction promote tumor progression? Oncogene. 2008;27(55):6920–6929. 19029934 10.1038/onc.2008.343 PMC2745643

[40] Ruiz-Martínez J , de la Riva P, Rodríguez-Oroz MC, et al Prevalence of cancer in Parkinson’s disease related to R1441G and G2019S mutations in LRRK2. Mov Disord. 2014;29(6):750–755. 24357540 10.1002/mds.25778

[41] Sugier PE , Lucotte EA, Domenighetti C, et al Investigation of shared genetic risk factors between Parkinson’s disease and cancers. Mov Disord. 2023;38(4):604–615. 36788297 10.1002/mds.29337 PMC10334300

[42] Driver JA . Understanding the link between cancer and neurodegeneration. J Geriatr Oncol. 2012;3(1):58–67.

[43] Driver JA . Inverse association between cancer and neurodegenerative disease: Review of the epidemiologic and biological evidence. Biogerontology. 2014;15(6):547–557. 25113739 10.1007/s10522-014-9523-2

[44] Morris LGT , Veeriah S, Chan TA. Genetic determinants at the interface of cancer and neurodegenerative disease. Oncogene. 2010;29(24):3453–3464. 20418918 10.1038/onc.2010.127 PMC3005561

[45] Ji J , Sundquist K, Sundquist J. Cancer incidence in patients with polyglutamine diseases: A population-based study in Sweden. Lancet Oncol. 2012;13(6):642–648. 22503213 10.1016/S1470-2045(12)70132-8

[46] Minhas PS , Jones JR, Latif-Hernandez A, et al Restoring hippocampal glucose metabolism rescues cognition across Alzheimer’s disease pathologies. Science. 2024;385(6711):eabm6131. 10.1126/science.abm6131 PMC12313320 39172838

