# Supplementary material for: HTT, ATXN1 and ATXN2 CAG triplet repeat sizes: exploring their role in the disease risk and cancer comorbidity in Parkinson’s disease
Source: Brain Commun. 2025 Feb 6;7(1):fcaf060. doi: 10.1093/braincomms/fcaf060 (PMC11837329; doi:10.1093/braincomms/fcaf060)
Supplement: fcaf060_Supplementary_Data [file fcaf060_supplementary_data.pdf]

Supplementary Table 1. Gene panel information for sequencing neurological disorders.

Name	Chromosome	Num_Amplicons	Total_Bases	Covered_Bases	Missed_Bases	Overall_Coverage
AARS	chr16	29	3907	3907	0	1
ABCD1	chrX	20	2338	2338	0	1
AFG3L2	chr18	25	3244	3244	0	1
AIFM1	chrX	22	2881	2881	0	1
ALS2	chr2	53	6702	6702	0	1
AP5Z1	chr7	28	3274	3274	0	1
APOE	chr19	11	1232	1232	0	1
APP	chr21	23	2545	2545	0	1
ATL1	chr14	22	2377	2377	0	1
ATP13A2	chr1	38	4069	4069	0	1
ATP7A	chrX	41	4723	4723	0	1
BSCL2	chr11	13	1499	1499	0	1
CACNA1A	chr19	73	8111	8111	0	1
CHMP2B	chr3	8	942	942	0	1
CYP27A1	chr2	14	2046	2046	0	1
CYP7B1	chr8	13	1821	1821	0	1
DCAF8	chr1	15	2394	2394	0	1
DCTN1	chr2	40	4169	4169	0	1
DHTKD1	chr10	30	3610	3590	20	0.9945
DNAJB2	chr2	12	1436	1436	0	1
DNAJC13	chr3	79	9482	9482	0	1
DNM2	chr19	26	2972	2972	0	1
DNMT1	chr19	50	5309	5309	0	1
DST	chr6	193	22941	22941	0	1
DYNC1H1	chr14	110	14721	14721	0	1
EGR2	chr10	10	1531	1531	0	1
EIF4G1	chr3	44	6424	6424	0	1
FA2H	chr16	11	1469	1469	0	1
FBXO38	chr5	37	4392	4392	0	1
FBXO7	chr22	17	2106	2106	0	1
FGD4	chr12	31	3664	3664	0	1
FGF14	chr13	9	1252	1252	0	1
FIG4	chr6	33	2954	2954	0	1
FUS	chr16	17	1731	1731	0	1
GARS	chr7	21	2390	2390	0	1
GBA	chr1	13	1721	1721	0	1
GDAP1	chr8	7	1137	1137	0	1
GIGYF2	chr2	41	5366	5366	0	1
GJB1	chrX	5	902	902	0	1
GJB3	chr1	6	823	823	0	1
GNB4	chr3	13	1473	1473	0	1
GRN	chr17	17	1902	1902	0	1
HARS	chr5	14	2050	2050	0	1
HK1	chr10	31	4016	4016	0	1
HSPB1	chr7	7	768	768	0	1
HSPB3	chr5	3	503	503	0	1
HSPB8	chr12	6	741	741	0	1
HSPD1	chr2	18	2272	2272	0	1
IGHMBP2	chr11	28	3732	3732	0	1
IKBKAP	chr9	39	4359	4359	0	1
INF2	chr14	36	4003	4003	0	1
ITPR1	chr3	68	8877	8877	0	1
KCNA1	chr12	9	1498	1498	0	1
KCND3	chr1	17	2318	2318	0	1
KIAA0196	chr8	40	4880	4880	0	1
KIF1A	chr2	66	7776	7776	0	1
KIF1B	chr1	71	9148	9148	0	1
KIF1C	chr17	35	4362	4362	0	1
KIF5A	chr12	42	4499	4499	0	1
L1CAM	chrX	39	4054	4054	0	1
LITAF	chr16	5	768	768	0	1
LMNA	chr1	18	2369	2369	0	1
LRRK2	chr12	86	8094	8093	1	0.9999

LRSAM1	chr9	27	2412	2412	0	1
MAPT	chr17	19	2471	2471	0	1
MARS	chr12	29	3753	3753	0	1
MED25	chr19	27	3144	3144	0	1
MFN2	chr1	21	2444	2444	0	1
MME	chr3	30	3353	3353	0	1
MPZ	chr1	10	1239	1239	0	1
MTMR2	chr11	21	2682	2682	0	1
NAGLU	chr17	17	2532	2532	0	1
NDRG1	chr8	21	1935	1935	0	1
NEFL	chr8	11	1882	1882	0	1
NGF	chr1	4	776	776	0	1
NIPA1	chr15	9	1240	1240	0	1
NTRK1	chr1	28	2703	2703	0	1
PARK2	chr6	15	1518	1518	0	1
PARK7	chr1	8	870	841	29	0.9667
PDK3	chrX	13	1852	1852	0	1
PINK1	chr1	14	2146	2146	0	1
PLA2G6	chr22	23	2581	2581	0	1
PLEKHG5	chr1	39	4699	4699	0	1
PLP1	chrX	9	904	904	0	1
PMP22	chr17	5	523	523	0	1
PRKCG	chr19	28	3087	3087	0	1
PRNP	chr20	5	812	812	0	1
PRPS1	chrX	8	1027	1027	0	1
PRX	chr19	31	4489	4484	5	0.9989
PSEN1	chr14	12	1504	1504	0	1
PSEN2	chr1	15	1447	1447	0	1
RAB39B	chrX	5	742	742	0	1
RAB7A	chr3	7	874	874	0	1
REEP1	chr2	9	1120	1120	0	1
SACS	chr13	82	13830	13830	0	1
SBF1	chr22	59	7732	7732	0	1
SBF2	chr11	66	7550	7550	0	1
SCN11A	chr3	49	6676	6676	0	1
SCN9A	chr2	51	6194	6194	0	1
SETX	chr9	61	8274	8274	0	1
SH3TC2	chr5	30	4717	4717	0	1
SIGMAR1	chr9	7	909	909	0	1
SLC2A1	chr1	14	1579	1579	0	1
SLC5A7	chr2	17	2143	2143	0	1
SNCA	chr4	5	673	673	0	1
SOD1	chr21	5	515	515	0	1
SPAST	chr2	24	2701	2701	0	1
SPG11	chr15	59	7732	7714	18	0.9977
SPG7	chr16	22	2714	2714	0	1
SPTBN2	chr11	72	8973	8973	0	1
SPTLC1	chr9	20	2379	2379	0	1
SPTLC2	chr14	19	2289	2289	0	1
SQSTM1	chr5	14	1723	1723	0	1
SURF1	chr9	10	1353	1348	5	0.9963
SYT2	chr1	13	1660	1660	0	1
TARDBP	chr1	10	1295	1295	0	1
TBK1	chr12	27	3190	3190	0	1
TFG	chr3	14	1553	1553	0	1
TGM6	chr20	24	2771	2771	0	1
TH	chr11	21	2287	2287	0	1
TREM2	chr6	9	1104	1104	0	1
TRIM2	chr4	20	3008	3008	0	1
TRPV4	chr12	21	2766	2766	0	1
TTBK2	chr15	34	4435	4435	0	1
VCP	chr9	21	2591	2591	0	1
VPS35	chr16	27	3241	3241	0	1
WNK1	chr12	68	10143	10143	0	1
YARS	chr1	16	2237	2237	0	1
ZFYVE26	chr14	66	9670	9670	0	1

Note: "Num_Amplicons" represents the number of amplification regions used for sequencing. "Total_Bases" indicates the total length of the targeted regions. "Covered_Bases" and "Missed_Bases" denote the number of bases successfully sequenced and those that were not covered, respectively. "Overall_Coverage" is expressed as a fraction of 1, representing the proportion of successfully sequenced bases relative to the total targeted region, with values close to 1 indicating near-complete coverage.

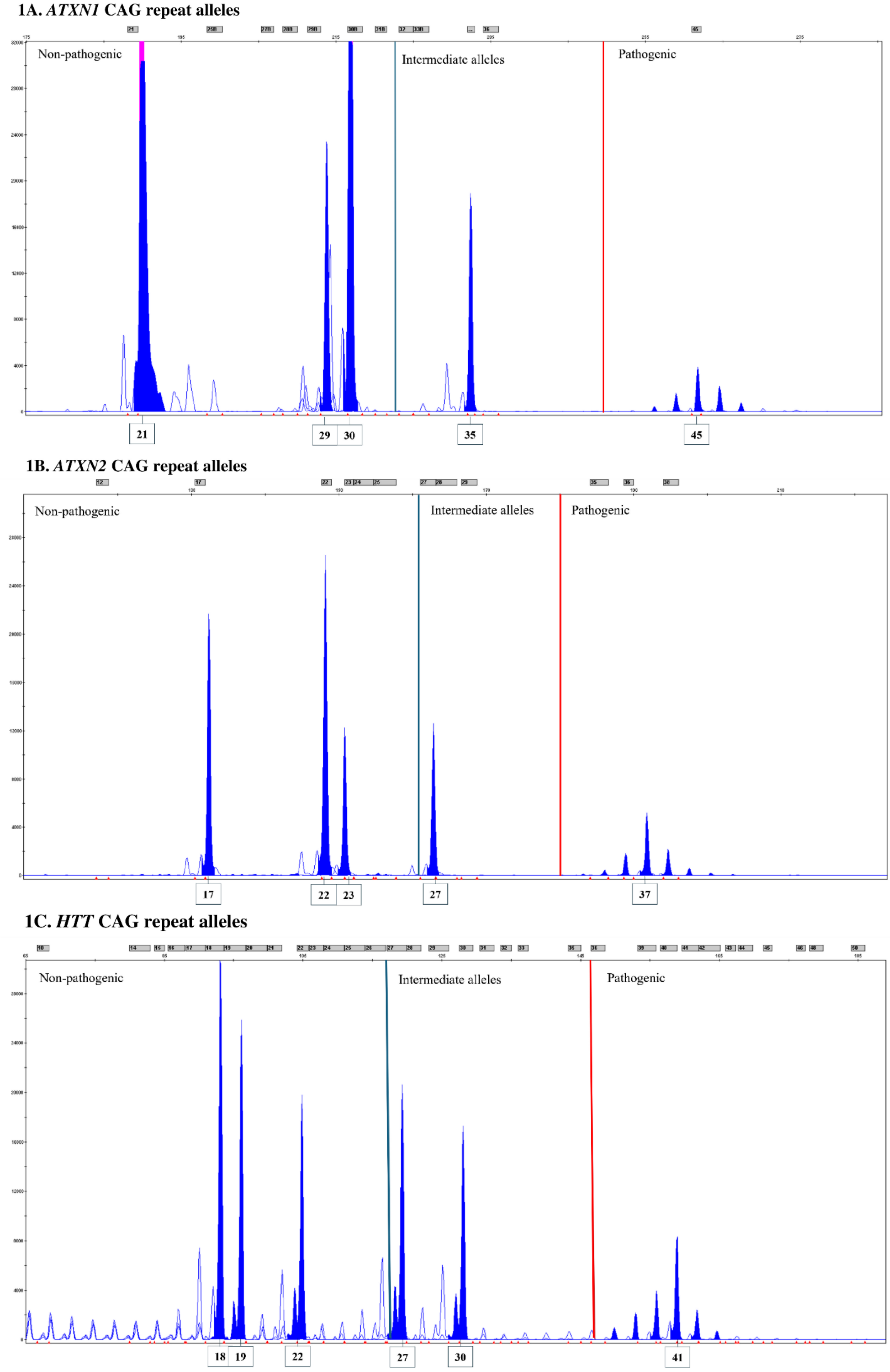

Supplementary figure 1. Electropherogram for detecting CAG repeat ranges in the HTT, ATXN1, and ATXN2 genes.

The electropherogram illustrates the detection of CAG repeat sizes for the HTT, ATXN1, and ATXN2 genes using standard genotyping software. Peaks in the electropherogram correspond to the fluorescence signal intensity for each repeat size, which is automatically detected and quantified by the software. Each peak's size corresponds to the CAG repeat length, as determined by comparing the migration distance to a size standard.

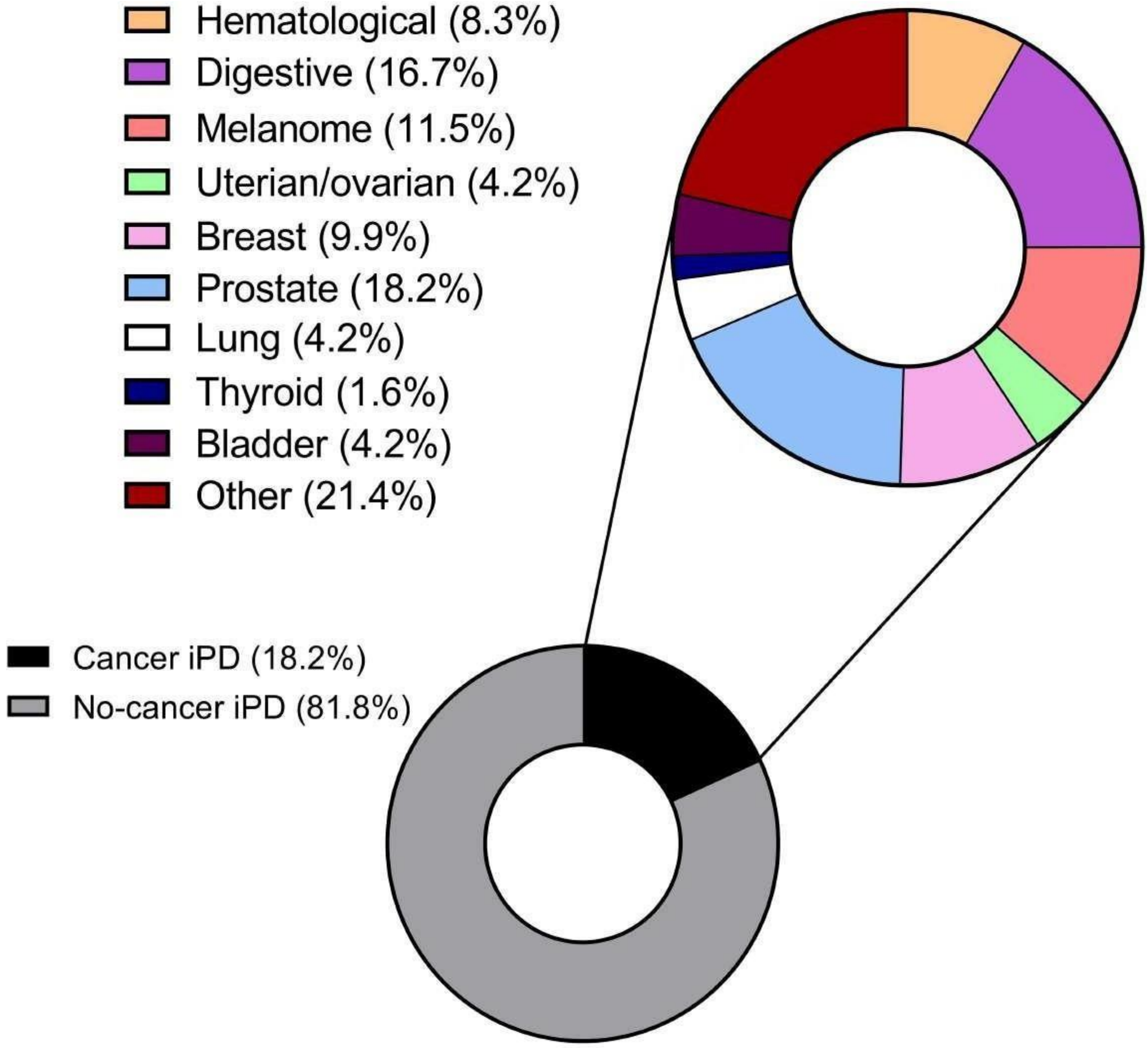

Supplementary figure 2. Cancer frequencies in the PD cohort. *The graphs show the relative frequency (%) of different types of cancer in the Parkinson's Disease cohort.*

Supplementary Table 2- Association between CAG repeat number and PD risk.

Gene						
HTT		**SE**	**Zvalue**	**P value**	**OR**	**95% CI**
	**HTT2**	0.114	-2.914	**0.004**	0.718	(0.572, 0.895)
	**HTT2^2**	0.003	2.561	**0.010**	1.007	(1.002, 1.012)
**Male model - HTT2**		0.170	-2.208	**0.027**	0.688	(0.493,0.966)
ATXN1		**SE**	**Zvalue**	**P value**	**OR**	**95% CI**
	**ATXN1-1**	1.135	0.783	0.434	2.432	(0.290,26.29)
	**ATXN1-1^2**	0.018	2.265	**0.024**	1.041	(1.001,1.078)
	**ATXNl-2**	1.173	2.310	**0.021**	15.023	(1.579,159.65)
	**ATXN1[1-2]**	0.040	-2.389	**0.017**	0.908	(0.837,0.981)
**Male model-*ATXN1***		**SE**	**Zvalue**	**P value**	**OR**	**95% CI**
	**ATXN1-1**	2.089	2.802	**0.005**	347.99	(7.523,2.63e+04)
	**ATXNl-2**	1.960	2.671	**0.008**	187.54	(5.076, 1.072e+04)
	**ATXN1[1-2]**	0.067	-2.706	**0.007**	0.834	(0.725,0.943)
**Female model - ATXN1-1^2**		0.002	2.632	**0.009**	1.004	(1.001,1.008)
ATXN2		**SE**	**Zvalue**	**P value**	**OR**	**95% CI**
	ATXN2-1	4.625	1.176	0.239	2.30.59	(0.128,4.30e+06)
	**ATXN2-1^2**	0.133	-3.641	**2.72e-04**	0.616	(0.451,0.770)
	**ATXN2-2**	4.431	-3.120	**0.002**	9.915e-07	(3.73e-11,3.35e-03)
	**ATXN2[1-2]**	0.201	3.122	**0.002**	1.874	(1.291,2.977)

To correctly evaluate where the interaction lies, we evaluate PD vs. controls; SE-Standard Error;OR-Odds Ratio; 95%CI-95% Confidence Interval
